# Loss of NDRG2 in liver microenvironment inhibits cancer liver metastasis by regulating tumor associate macrophages polarization

**DOI:** 10.1038/s41419-018-0284-8

**Published:** 2018-02-14

**Authors:** Mengyang Li, Xiaofeng Lai, Ying Zhao, Yuan Zhang, Minghui Li, Danxiu Li, Jing Kong, Yong Zhang, Pengyu Jing, Huichen Li, Hongyan Qin, Liangliang Shen, Libo Yao, Jipeng Li, Kefeng Dou, Jian Zhang

**Affiliations:** 1 State Key Laboratory of Cancer Biology, Department of Biochemistry and Molecular Biology, Fourth Military Medical University, Xi’an, China; 2 Department of Hepatobiliary and Pancreas Surgery, Xijing Hospital Fourth Military Medical University, Xi’an, China; 3 State Key Laboratory of Cancer Biology, Institute of Digestive Diseases, Xijing Hospital The Fourth Military Medical University, Xi’an, China; 4 Department of Orthopedics, Xijing Hospital Fourth Military Medical University, Xi’an, China; 5 Department of Thyroid, Breast and Vascular Surgery, Xijing Hospital Fourth Military Medical University, Xi’an, China; 6 Department of Pulmonary Medicine, Xijing Hospital Fourth Military Medical University, Xi’an, China; 7 Department of Thoracic Surgery, Tangdu Hospital Fourth Military Medical University, Xi’an, China; 8 Department of Medical Genetics and Developmental Biology, Fourth Military Medical University, Xi’an, China

## Abstract

The liver is the predominant metastatic site for several types of malignancies. Tumor-associated macrophages (TAMs) in the liver play crucial roles in the metastasis process. Shifting tumor-promoting M2-like TAMs toward the M1-like phenotype, which exerts tumor suppressor functions via phagocytosis and the secretion of inhibitory factors, may be a potential therapeutic strategy for liver cancer metastasis treatment.

We first cloned *NDRG2* (N-myc downstream-regulated gene 2) and verified its tumor suppressor role in multiple solid tumors, including colorectal cancer and hepatocellular carcinoma. However, its role in the tumor-associated liver microenvironment, especially in TAMs, has not been illustrated. By establishing a liver cancer metastasis model in wild-type (WT) and *Ndrg2* knockout (*Ndrg2−/−*) mice, we found that the loss of the tumor suppressor *Ndrg2* in liver microenvironment significantly suppressed the growth of liver colonies. In addition, this process was accompanied by a higher proportion of M1-like TAM infiltration in *Ndrg2−/−* mice. Interestingly, bone marrow (BM) transplantation revealed that BM-derived macrophages (BMDMs) rather than liver resident Kupffer cells were responsible for the inhibitory effect. We further demonstrated that loss of *Ndrg2* influenced TAM polarization via the NF-κB pathway. Inhibition of IκBα phosphorylation in cancer cell-conditioned medium-stimulated BMDMs decreased M1 marker expression in *Ndrg2−/−* macrophages. Finally, in vitro, invasion, migration, and proliferation assays confirmed that NF-κB participated in the tumor suppressor function of *Ndrg2−/−* macrophages. Collectively, our findings highlight the role of NDRG2 in the regulation of TAM polarization and its function in promoting cancer liver metastasis.

## Introduction

Malignancy in the liver threatens the lives of patients. The liver is the site of primary liver cancer as well as the predominant metastatic site for several kinds of cancer, such as colorectal cancer (CRC), lung cancer, melanoma, and gastric carcinoma^1,2^. The generation and development of metastatic lesions is responsible for the high mortality associated with these diseases. Cancer liver metastasis is a complex process that includes several major steps: invasion and penetration of microvessels; cell survival in the circulation and establishment in the liver; formation of a metastatic niche; and tumor cell expansion^3^. Primary tumors can release a large number of cancer cells into the circulation, while only a small proportion of these cells can survive in the liver. During this process, in addition to genetic changes that occur in cancer cells, increasing evidence has confirmed that the participation of the tumor-associated microenvironment is indispensable for this process^4,5^.

The tumor-associated liver microenvironment is composed of hepatocytes, Kupffer cells (KC), hepatic sinusoidal endothelial cells, hepatic stellate cells (HSCs) and recruited immune cells, such as T cells, NK cells, bone-marrow- derived macrophages (BMDMs), etc^6^. The fate of tumor cells can be determined by the interaction of these components in the liver sinusoid. Macrophages are a prominent component of the immune cells recruited; however, their functions under different conditions seem to be highly plastic^7^. Previous studies suggest that liver metastasis-associated macrophages mainly exhibit a tumor-promoting phenotype. Selectively programming or re-educating macrophages toward a tumor-suppressor phenotype can be a potential therapeutic strategy^3^.

Our previous data showed that N-myc downstream-regulated gene 2 (*NDRG2*) was a candidate tumor suppressor in several kinds of cancers^8–10^. NDRG2 exerts its tumor suppressor function by influencing cancer cell proliferation and metabolism and suppressing angiogenesis^11,12^. In CRC, the expression of NDRG2 was significantly decreased in tumors compared with that in normal tissues, and patients with relatively high NDRG2 expression levels tended to have better overall survival^8^. Despite its role in cancer cells, the exact function of NDRG2 in the liver metastasis-associated microenvironment, especially in metastasis-associated macrophages, has not been investigated.

Here, we established a cancer liver metastasis model in wild-type and *Ndrg2* knockout (*Ndrg2−/−*) mice and found that the *Ndrg2−/−* liver microenvironment significantly suppressed the growth of liver colonies with infiltration of a higher proportion of M1-like tumor-associated macrophages (TAMs). We further demonstrated that the influence of loss of *Ndrg2* on TAM polarization was dependent on the NF-κB pathway. Collectively, our study highlights the important role of NDRG2 in the regulation of TAM polarization and its function in promoting cancer liver metastasis.

## Results

### Loss of Ndrg2 inhibits liver metastasis

**Fig. 1:**
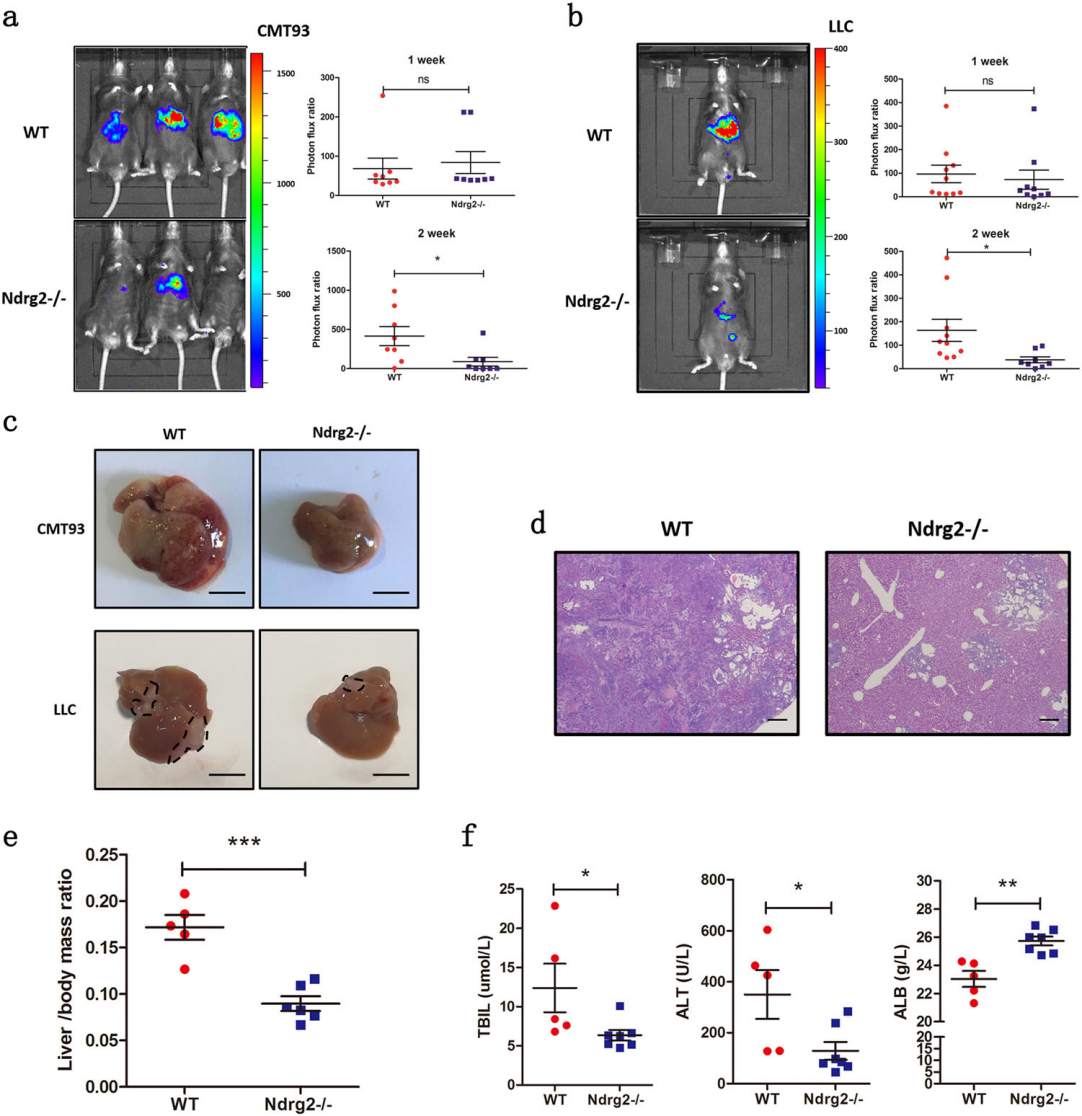
**Loss of Ndrg2 inhibits liver cancer metastasis**. **a** Bioluminescence on days 7 and 14 post-CMT93-luciferase cell injection. Quantification of the photon flux ratio per mouse at each time point. *n* = 8 mice per group. **b** Bioluminescence on days 7 and 14 post-LLC-luciferase cell injection. Quantification of the photon flux ratio per mouse at each time point. *n* = 9−10 mice per group. **c** Gross examination of CMT93 and LLC cell-induced liver metastasis in WT or *Ndrg2−/−* mice. Bar = 1 cm. **d** HE staining of metastatic foci after intra-splenic injection of CMT93 cells in WT and *Ndrg2−/−* mice. Bar = 500 μm. **e** Liver/body weight ratio measured 14 days after CMT93 metastasis model establishment. *n* = 5−6 mice per group. **f** Concentration of the WT or *Ndrg2−/−* mouse serum liver function markers TBIL, ALT, and ALB on day 14 after CMT93 injection. *n* = 5−7 mice per group. The data are presented as the mean ± SEM *p < 0.05, **p < 0.01, and ***p < 0.001

*NDRG2* has long been regarded as a candidate tumor suppressor gene. It exerts its tumor suppressor function by inhibiting tumor cell proliferation, migration, and angiogenesis via several pathways. However, previous research has not focused on its function in tumor-associated microenvironments. To determine the role of NDRG2 in the liver microenvironment during cancer liver metastasis, we established CMT93 and Lewis lung carcinoma (LLC) cell liver metastasis model. Luciferase-expressing CMT93 murine colorectal cells or LLC murine lung carcinoma cells were injected into the spleen of WT and *Ndrg2−/−* mice, which enabled efficient cancer cell dissemination to the liver. On the 7th and 14th day post-injection, in vivo bioluminescence was used to monitor the metastatic lesions in the liver. On day 14, the increase in bioluminescence intensity was remarkably suppressed in *Ndrg2−/−* mice livers compared with that in the WT mice livers, although they showed essentially the same level of bioluminescence on day 7 (Fig. 1a, b). We further confirmed this phenotype in an intra-hepatic injection model and subcutaneous injection model (Supplement Fig. 1a and b). On day 14, mice were killed, and the liver/body mass ratio was measured. WT mice liver weight increased significantly compared with that of normal livers (Fig. 1e, Supplement Fig. 1c), with diffuse tumor foci throughout the liver. In contrast, *Ndrg2−/−* mice livers showed fewer tumor foci (Fig. 1c). Histopathology revealed that the biological structure of WT mice livers was destroyed by the liver colonies, whereas the *Ndrg2−/−* mice livers retained a normal structure and showed a significant reduction in the metastatic lesion (Fig. 1d). We tested several liver function markers, namely, ALT, TBIL, and ALB serum levels, and we found that ALT and TBIL were significantly elevated in the WT group compared with those in the *Ndrg2−/−* group, whereas *Ndrg2−/−* mice had higher ALB levels than did WT mice, revealing that *Ndrg2−/−* mice had better liver function reserves (Fig. 1f). Collectively, these data suggest that the loss of NDRG2 in the liver microenvironment suppresses liver cancer colonies and preserves liver function.

### Loss of Ndrg2 influences tumor-associated macrophage polarization at liver metastasis sites

**Fig. 2:**
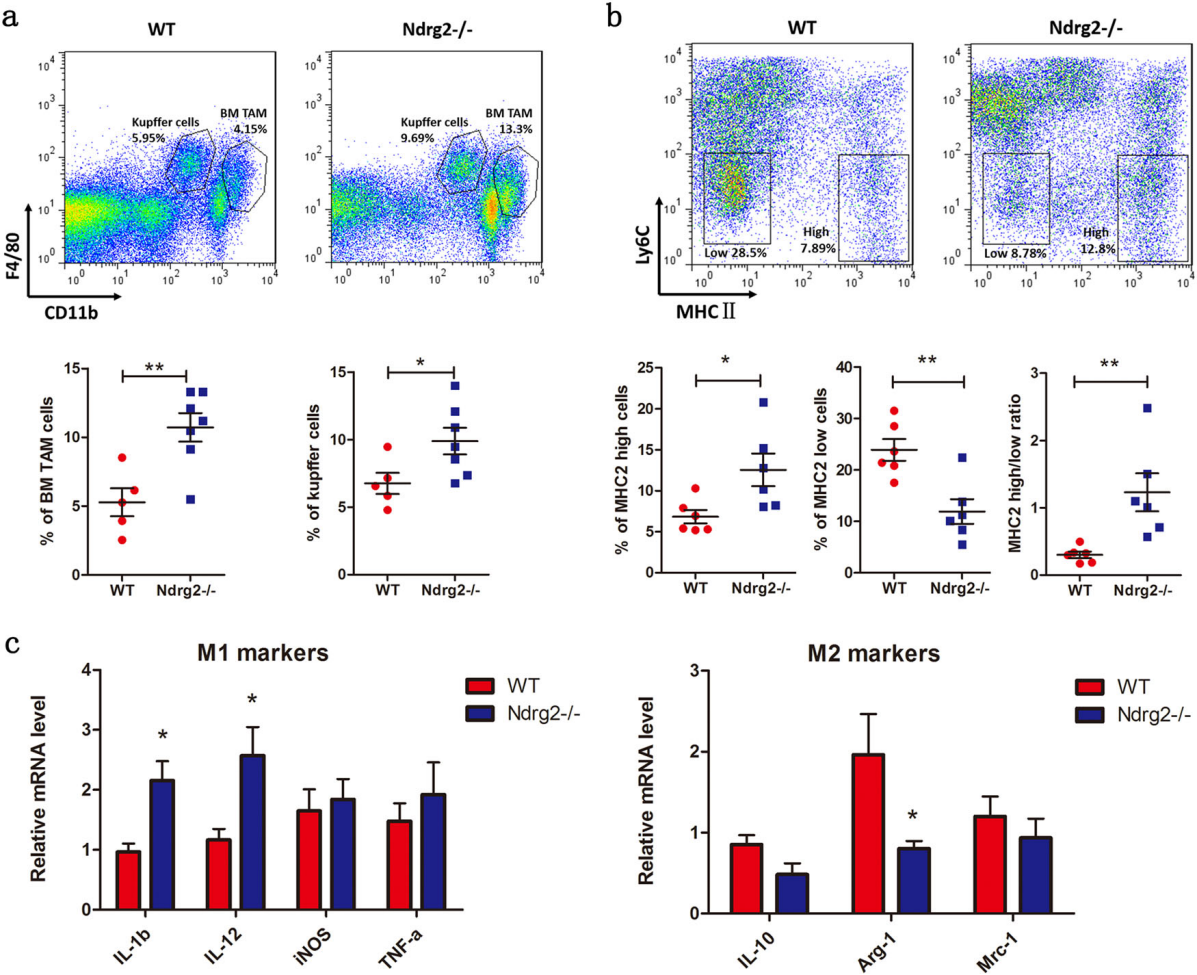
**Loss of Ndrg2 influences tumor-associated macrophage polarization**. **a** Flow cytometry analysis of macrophages infiltrated into WT or *Ndrg2−/−* mouse metastatic lesions. Among the CD45^+^ cells, BM-derived TAMs and Kupffer cells were gated and analyzed. *n* = 5−7 mice per group. **b** Flow cytometry analysis of macrophage subpopulations based on Ly6C and MHCII expression. Among F4/80^+^ macrophages, Ly6C^lo^ MHCII^hi^ M1-like TAMs and Ly6C^lo^ MHCII^lo^ M2-like TAMs were gated and analyzed. *n* = 6 mice per group. **c** At 14 days after metastasis model establishment, macrophages in WT or *Ndrg2−/−* mouse metastatic lesions were isolated using magnetic beads, and q-PCR was used to test the indicated markers. *n* = 3 mice per group. The results are presented as the mean ± SEM *p < 0.05; **p < 0.01

Immune cells play a pivotal role in the formation of the tumor-associated microenvironment. We separated metastatic liver lesions. Flow-cytometry reveals that there were no differences in the proportions of T helper cells (CD3^+^ CD4^+^) or cytotoxic T lymphocytes (CD3^+^ CD8^+^), NK cells, M-MDSCs, G-MDSCs, and granulocytes between WT and *Ndrg2−/−* mice (Supplement Fig. 2b). B cells, KC and BMDMs showed significantly increased infiltration (Fig. 2a, Supplement Fig. 2a and b). Because TAM polarization influences proliferation, migration, invasion, and angiogenesis both in the primary tumor and in metastatic foci, we intended to clarify the polarization phenotype of the macrophages that had accumulated at the liver metastasis site. A previous study used Ly6C and MHCII to classify TAMs into several subpopulations^13,14^. Among these populations, Ly6C^lo^MHCII^hi^ TAMs are M1-like macrophages and Ly6C^lo^MHCII^lo^ TAMs are M2-like macrophages. Using this categorization, we found that the macrophages that had infiltrated into *Ndrg2−/−* mice liver metastatic lesions had a higher proportion of the Ly6C^lo^MHCII^hi^ tumor inhibitory subtype and a lower proportion of the Ly6C^lo^MHCII^lo^ tumor-promoting subtype (Fig. 2b). We then isolated the infiltrated macrophages from the WT and *Ndrg2−/−* mice metastatic lesions and tested the expression levels of several polarization markers. The results showed that IL-1β and IL-12 expression levels were increased in the *Ndrg2−/−* group together with a decrease in Arg-1 expression levels, revealing a tumor-suppressor phenotype (Fig. 2c). Overall, the observed tumor growth inhibition in *Ndrg2−/−* metastatic lesions was accompanied by an increased proportion of M1-like tumor suppressor macrophages.

### Loss of NDRG2 in bone-marrow-derived macrophages contributed to the inhibitory microenvironment during liver cancer metastasis

**Fig. 3:**
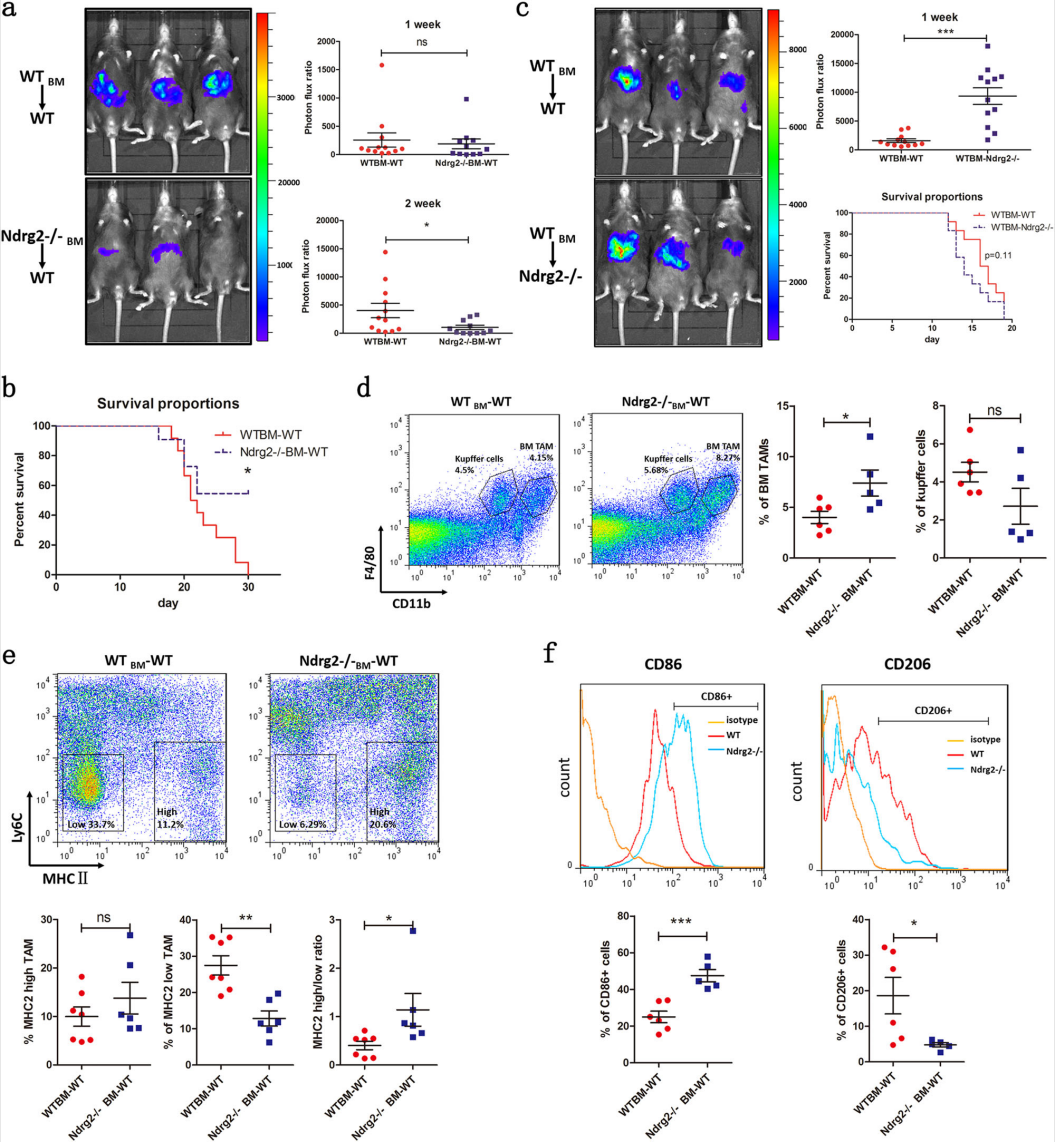
**Loss of Ndrg2 in BM-derived cells contributes to an inhibitory microenvironment**. **a** Recipient WT mice were lethally irradiated and transplanted with WT or *Ndrg2−/−* BMs. After reconstruction, the metastasis model was established. Bioluminescence was measured on days 7 and 14. The data were quantified by analyzing the photon flux ratio in the liver area. *n* = 11−12 mice per group. **b** Overall survival of *Ndrg2−/−*_BM_-WT or WT_BM_-WT reconstructed mice in the metastasis model. **c** Recipient WT or *Ndrg2−/−* mice were lethally irradiated and transplanted with WT BM. Bioluminescence was measured on day 7. Overall survival was observed. *n* = 12 per group. **d** Flow cytometry analysis of macrophages infiltrated into *Ndrg2−/−*_BM_-WT or WT_BM_-WT mouse metastatic lesions. BM-derived TAMs and Kupffer cells were gated and analyzed. *n* = 5−6 mice per group. **e** Flow cytometry analysis of macrophage subpopulations based on Ly6C and MHCII expression. Ly6C^lo^ MHCII^hi^ M1-like TAMs and Ly6C^lo^ MHCII^lo^ M2-like TAMs were gated and analyzed. *n* = 6−7 mice per group. **f** Flow cytometry analysis of macrophage polarization based on CD86 and CD206 expression. The percentages of CD86^+^ or CD206^+^ macrophages were calculated. *n* = 5−6 mice per group. The data are presented as the mean ± SEM *p < 0.05, **p < 0.01, and ***p < 0.001

To further explore whether the loss of NDRG2 in tumor-infiltrating macrophages could contribute to the decreased tumor metastasis in *Ndrg2−/−* mice, we transplanted bone marrow (BM) from syngeneic WT or *Ndrg2−/−* mice into lethally irradiated WT recipients. After stable engraftment, we established a tumor liver metastasis model in these BM-reconstructed mice. Bioluminescence revealed that on day 7, there was no significant difference in metastasis formation between *Ndrg2−/−*_BM_ → WT mice and WT_BM_ → WT mice; however, on day 14, metastasis in *Ndrg2−/−*_BM_ → WT mice was inhibited compared with that in WT_BM_ → WT mice, as quantified by a significantly lower bioluminescence intensity (Fig. 3a). In addition, *Ndrg2−/−*_BM_ → WT mice also showed an extended overall survival compared with that of WT_BM_ → WT mice (Fig. 3b). Taken together, these data indicated that *Ndrg2−/−* BM contributed to the inhibition of liver metastasis.

We then conducted WT_BM_ → *Ndrg2−/−* and WT_BM_ → WT mice BM transplantation. Surprisingly, we found that WT_BM_ → *Ndrg2−/−* mice showed more severe liver metastasis formation than WT_BM_ → WT mice, and half of the WT_BM_ → *Ndrg2−/−* mice survived less than 14 days. Regarding overall survival, although not significant, WT_BM_ → *Ndrg2−/−* mice tended to have poorer survival than WT_BM_ → WT mice (*p* = 0.11) (Fig. 3c). These data indicated that in *Ndrg2−/−* mice, aside from the BM-derived cells, other components of the liver environment, including KC, generally tended to show a tumor-promoting phenotype.

Flow cytometry analysis of the metastatic model in *Ndrg2−/−*_BM_ → WT mice and WT_BM_ → WT mice revealed that the proportion of infiltrated BMDMs in *Ndrg2−/−*_BM_ → WT mice was higher than that in the control group, while the proportion of KC did not differ between these two groups (Fig. 3d). Moreover, infiltrated macrophages in *Ndrg2−/−*_BM_ → WT mice tended to be M1-like compared with those in WT_BM_ → WT mice, with a lower proportion of the Ly6C^lo^MHCII^lo^ tumor-promoting subtype and a higher Ly6C^lo^MHCII^hi^ / Ly6C^lo^MHCII^lo^ ratio (Fig. 3e). CD86 and CD206 staining also revealed a higher proportion of CD86^+^ and lower proportion of CD206^+^ macrophages in *Ndrg2−/−*_BM_ → WT mice than in WT_BM_ → WT mice (Fig. 3f).

**Fig. 4:**
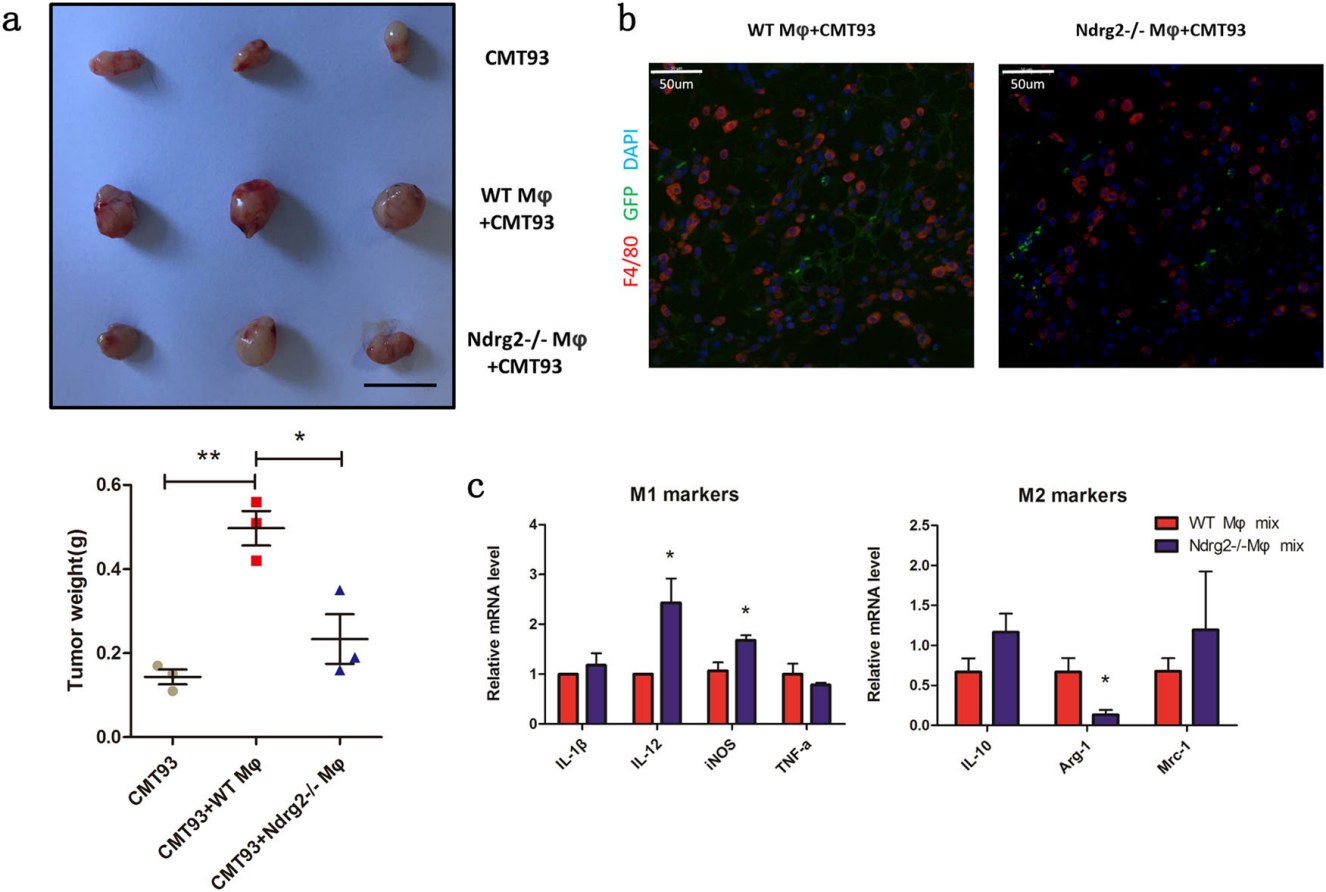
**Ndrg2−/− BMDMs inhibit tumor growth.** **a** WT mice were subcutaneously inoculated with 2×10^6^ CMT93 cells, 2×10^6^ CMT93 cells mixed with 5×10^5^ WT macrophages or 2×10^6^ CMT93 cells mixed with 5×10^5^ *Ndrg2−/−* macrophages. At 21 days after injection, the mice were killed, and the tumors were weighed. *n* = 3 mice per group. **b** Immunofluorescence analysis of F4/80 (red), GFP (green) and DAPI (blue) staining of tumor tissues. Scale bars = 50 μm. **c** At 21 days after the indicated cell inoculation (2×10^6^ CMT93 cells mixed with 5×10^5^ WT macrophages or 2×10^6^ CMT93 cells mixed with 5×10^5^ *Ndrg2−/−* macrophages), macrophages were isolated using magnetic beads, and q-PCR was used to test the indicated markers. *n* = 3 mice per group. The results are presented as the mean ± SEM *p < 0.05; **p < 0.01

To confirm that the loss of *Ndrg2* in BMDMs inhibited tumor growth, we established a mixed model of tumor cells and macrophages. In this model, 2×10^6^ CMT93 cells were mixed with 5×10^5^ WT or *Ndrg2−/−* BMDMs and subcutaneously injected into WT host mice. At 21 days after model establishment, tumors were removed and weighed. We found that WT macrophages mixed with CMT93 cells (WT_mφ_ MIX) significantly promoted tumor growth compared with *Ndrg2−/−* macrophages (*Ndrg2−/−*_mφ_ MIX) and tumors without the macrophage premix (CMT93), while *Ndrg2−/−* macrophages showed an attenuated tumor-promoting potential (Fig. 4a). We then used GFP mice as the host mice so that the host-derived macrophages were all GFP^+^, while the premixed macrophages were GFP^**−**^. Twenty-one days after model establishment, the mice were sacrificed. Immunofluorescence showed that nearly all the F4/80^+^ macrophages were GFP^+^ host-derived macrophages (Fig. 4b). GFP^−^premixed macrophages cannot survive for such a long period. We then isolated the F4/80^+^ macrophages with magnetic beads, and q-PCR was performed to examine the polarization marker levels. IL-12 and iNOS showed a significant increase in the *Ndrg2−/−*_mφ_ MIX group, while Arg-1 was reduced (Fig. 4c), indicating that premixed *Ndrg2−/−* macrophages not only tended to polarize toward an M1-like phenotype and suppress tumor growth but also could re-educate recruited macrophages toward a tumor-suppressor phenotype.

### Ndrg2 deficiency influences the macrophages polarization in vitro

**Fig. 5:**
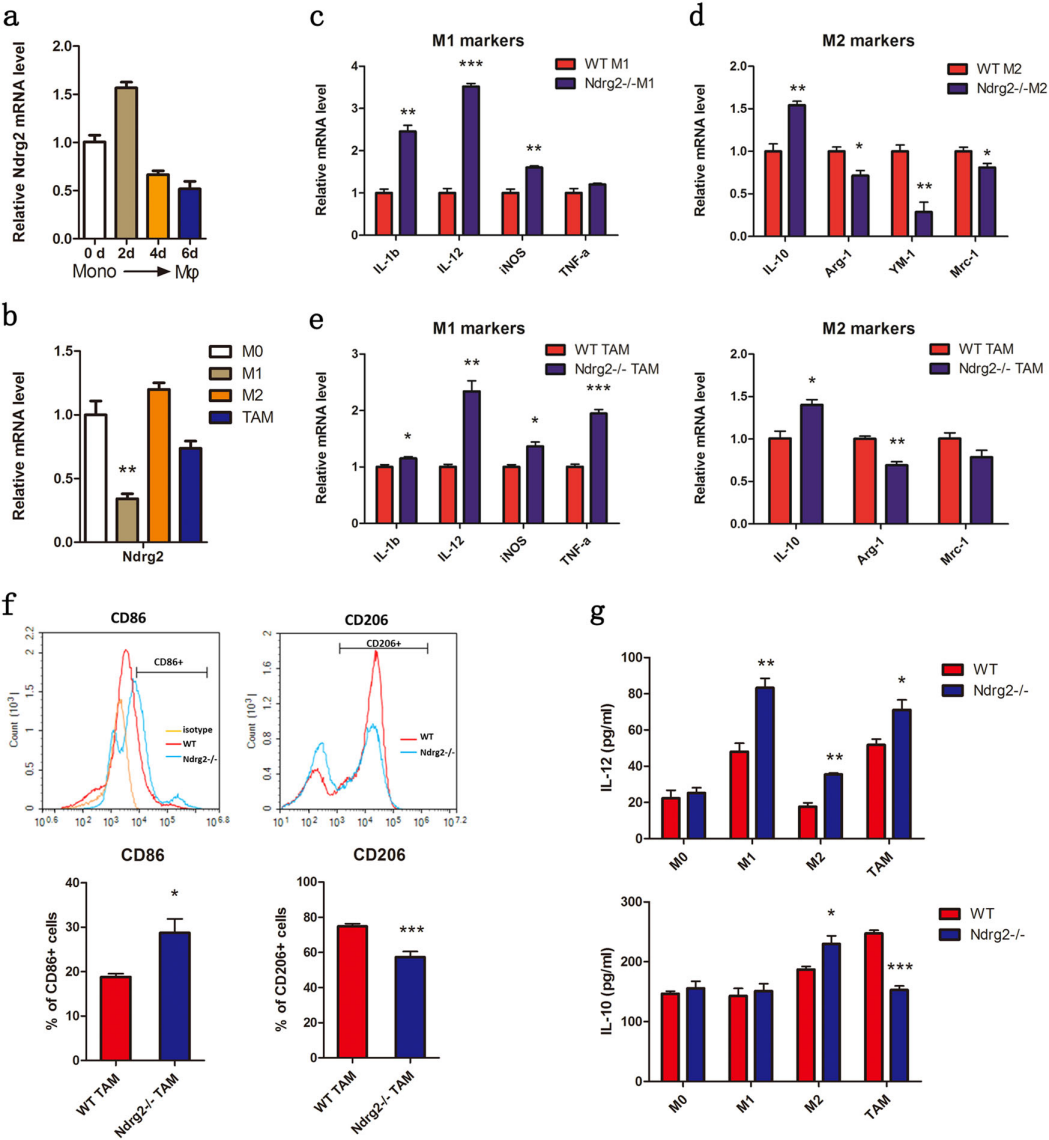
**Ndrg2 influences M1-like macrophage polarization in vitro.** **a** Relative expression changes in *Ndrg2* during the maturation process of monocytes toward macrophages. Monocytes/macrophages were isolated using magnetic beads and tested via q-PCR on days 0, 2, 4, and 6. **b** Relative expression of *Ndrg2* in M0, M1, and M2 macrophages and TAMs determined by q-PCR. *n* = 3 per group. **c**, **d** Relative expression of M1 and M2 markers in WT or *Ndrg2−/−* M1 and M2 BMDMs determined by q-PCR. *n* = 3 per group. **e** Relative expression of M1 and M2 markers in WT or *Ndrg2−/−* TAMs (induced by CMT93 cell-conditioned medium) determined by q-PCR. *n* = 3 per group. **f** CD86 and CD206 expression in CMT93 cell-conditioned medium-induced WT or *Ndrg2−/−*TAMs was analyzed using flow cytometry. *n* = 3 per group. **g** IL-12 and IL-10 concentrations in WT or *Ndrg2−/−* M0, M1, and M2 macrophages and TAM culture medium tested via ELISA. *n* = 3 per group. The results are presented as the mean ± SEM *p < 0.05, **p < 0.01, and ***p < 0.001

To clarify the possible role played by *Ndrg2* in macrophage polarization, we isolated BM cells from WT mice and induced monocytes to develop into mature macrophages with GM-CSF for 6 days. During this process, we used magnetic beads to isolate WT CD11b^+^ monocytes/macrophages on days 0, 2, 4, and 6 and tested their *Ndrg2* expression levels. The q-PCR results revealed elevated *Ndrg2* expression for a short period during the maturation of monocytes to macrophages (day 2) followed by a decrease on days 4 and 6 (Fig. 5a). BMDMs were treated with LPS and IFN-γ to induce M1 macrophages or with IL-4 to induce M2 macrophages. CMT93 cell-conditioned medium was used to induce BMDMs toward TAMs. *Ndrg2* expression was significantly decreased in induced M1 macrophages and slightly decreased in TAMs, with no significant change in M2 macrophages (Fig. 5b). All these results suggest that *Ndrg2* may be involved in macrophage maturation and M1 polarization.

*Ndrg2−/−* M1 macrophages showed a significant increase in M1-related markers, such as IL-Iβ, IL-12, and iNOS, compared with that in WT M1 macrophages (Fig. 5c). Except for IL-10, the M2-related markers (arginase-1, Mrc-1, and YM-1) decreased in *Ndrg2−/−* M2 macrophages (Fig. 5d). In addition, *Ndrg2−/−* TAMs appeared to have an IL-Iβ-, IL-12-, and TNF-α-high and arginase-1-low M1-like phenotype (Fig. 5e). Flow cytometry showed that *Ndrg2−/−* TAMs expressed higher levels of CD86 and lower levels of CD206 markers (Fig. 5f). ELISA also revealed that IL-12 was upregulated in *Ndrg2−/−* M1 and M2 macrophages and TAMs and that IL-10 was decreased in *Ndrg2−/−* TAMs (Fig. 5g). Taken together, the loss of NDRG2 induced macrophages toward a pro-inflammatory and tumor-suppressor phenotype.

### Loss of Ndrg2 influences macrophage polarization through the activation of NF-κB signaling

**Fig. 6:**
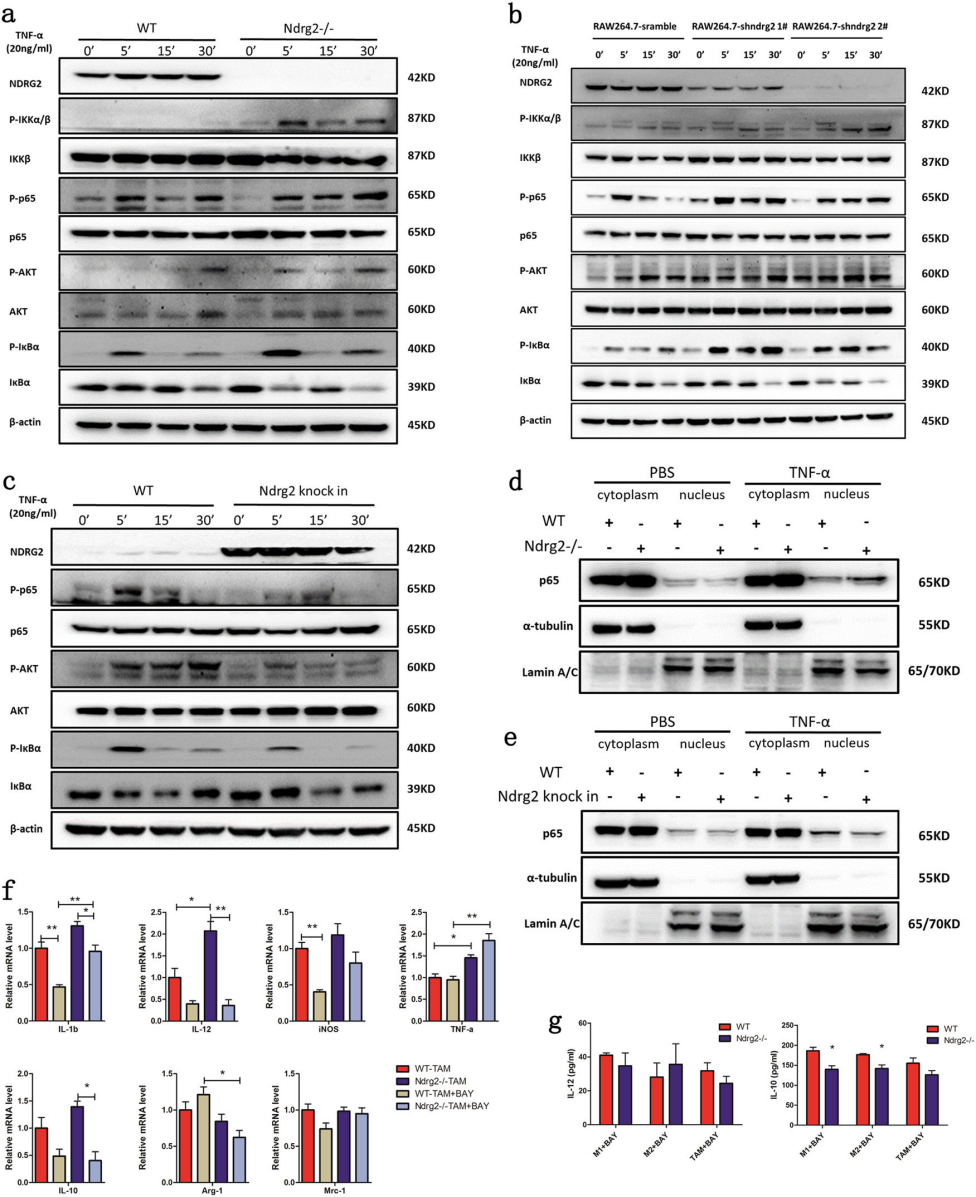
**Loss of Ndrg2 influences macrophage polarization through the activation of NF-κB signaling.** **a** WT or *Ndrg2−/−* BMDMs were treated with 20 ng/ml TNF-α for the indicated times, and western blotting was used to test the protein expression level as indicated. **b** RAW 264.7-scramble, RAW 264.7-shndrg2^1#^, and RAW 264.7-shndrg2^2#^ macrophages were treated with 20 ng/ml TNF-α for the indicated times, followed by western blot analysis. **c** WT or *Ndrg2 knock-in* BMDMs were treated with 20 ng/ml TNF-α and subjected to western blot analysis. **d** WT or *Ndrg2−/−* BMDMs were treated with or without TNF-α for 4 h. Cytoplasmic and nuclear proteins were separated. Western blotting was used to evaluate p65 nuclear translocation. **e** WT or *Ndrg2 knock-in* BMDMs were treated with or without TNF-α for 4 h. Western blotting was used to evaluate p65 expression in the cytoplasm and nucleus. **f** WT or *Ndrg2−/−* BMDMs were pretreated with DMSO or BAY-11-7082 for 4 h, followed by treatment with CMT93 cell-conditioned medium for 24 h. M1 and M2 markers were analyzed by q-PCR. *n* = 3 per group. **f** WT or *Ndrg2−/−* BMDMs were pretreated with BAY-11-7082 for 4 h and then induced toward M1, M2, and TAM phenotypes. The culture medium was collected after cultured in serum-free medium for 24 h. IL-12 and IL-10 concentrations were tested via ELISA. *n* = 3 per group. The results are presented as the mean ± SEM *p < 0.05; **p < 0.01

To determine the possible pathways involved in these phenotypes, we treated WT and *Ndrg2−/−* BMDMs with CMT93 cell-conditioned medium for 24 h and evaluated differences in the mRNA expression of 84 cancer immuno-crosstalk-associated genes (Supplement Fig. 3a). We found that among the eight genes that were upregulated twofold, four genes were related to the NF-κB pathway (Supplement Table 1). We then used western blotting to examine NF-κB pathway activation in BMDMs or RAW 264.7 macrophages. After stimulation with TNF-α in WT and *Ndrg2−/−* BMDMs, the phosphorylation status of IKKα/β, p65, and IκBα together with IκBα degradation were enhanced and prolonged in *Ndrg2−/−* BMDMs. A previous study revealed that NF-κB pathway activation is related to AKT phosphorylation. We then examined the phosphorylation status of AKT and found that *Ndrg2* deficiency resulted in enhancement of AKT phosphorylation (Fig. 6a). These results were also confirmed in RAW 264.7-scramble, RAW 264.7-sh Ndrg2 1^#^, and RAW 264.7-sh Ndrg2 2^#^ cells (Fig. 6b). We then examined the influence of *Ndrg2* overexpression on NF-κB pathway activation. BMDMs from WT and *Ndrg2 knock-in* mice were isolated. After stimulation with TNF-α, the phosphorylation of AKT, p65, and IκBα coupled with IκBα degradation was suppressed in *Ndrg2 knock-in* BMDMs compared with that in the WT group (Fig. 6c). We then separated the cytoplasm and nuclear protein from PBS- or TNF-α-treated BMDMs. Nuclear translocation of p65 was significantly enhanced in TNF-α-treated *Ndrg2−/−* BMDMs (Fig. 6d), whereas this process was diminished in *Ndrg2 knock-in* BMDMs (Fig. 6e). Bay-11-7082 has been reported to be an IκBα phosphorylation inhibitor, which suppresses p65 phosphorylation and nuclear translocation. The q-PCR results revealed that *Ndrg2−/−* TAMs had higher expression levels of M1-related genes than WT TAMs, but this phenotype was abolished by Bay-11-7082 (Fig. 6f). ELISA revealed that Bay-11-7082 treatment reduced IL-12 expression in WT and *Ndrg2−/−* M1 and M2 macrophages and in TAMs to the same level. Surprisingly, IL-10 also showed a slight decrease (Fig. 6g). Collectively, these data suggested that the loss of *Ndrg2* resulted in NF-κB pathway activation, which led to macrophage polarization toward an M1-like phenotype.

### The tumor-suppressor phenotype of Ndrg2*−*/*−* macrophages is reversed by blocking NF-κB signaling in vitro

**Fig.7:**
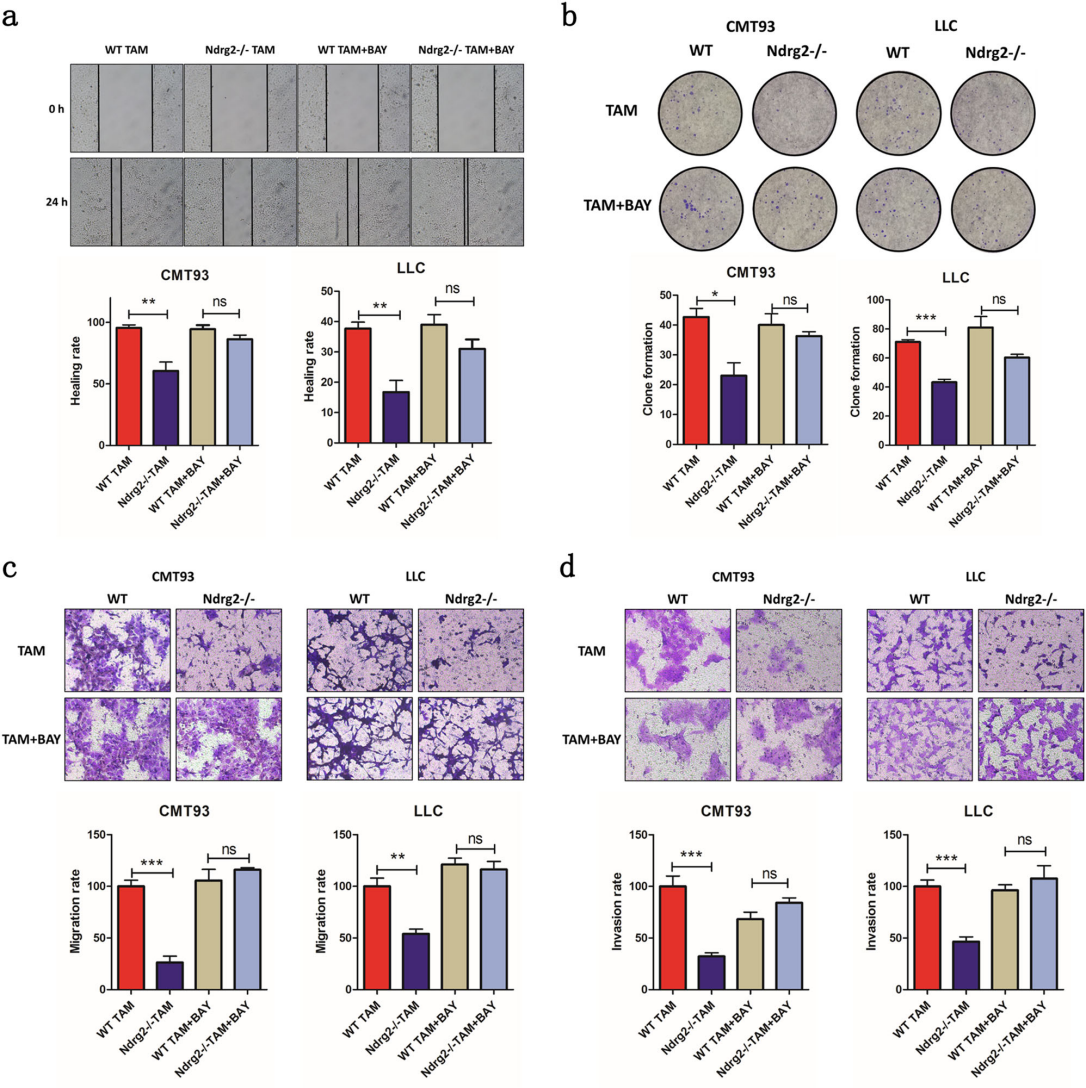
**The tumor-suppressor phenotype of Ndrg2−/− macrophages is reversed by blocking NF-κB signaling in vitro.** **a** A transwell co-culture system was used in the wound-healing assay. CMT93 cells were seeded into the lower chamber until complete fusion, and then, the scratch was generated. WT or *Ndrg2−/−* TAMs and TAMs pretreated with BAY-11-7082 were seeded into the upper chamber. After 24 h of co-culture, the scratch was imaged, and the fusion percentage was calculated. *n* = 3 per group. **b** Two hundred CMT93 or LLC cells were seeded into the plate and cultured with conditioned medium from WT or *Ndrg2−/−* TAMs and TAMs pretreated with BAY-11-7082. Colonies were stained and quantified. *n* = 3 per group. **c** CMT93 or LLC cells were seeded into the transwell upper chamber with WT or *Ndrg2−/−* TAMs, and TAMs pretreated with BAY-11-7082 were seeded into the lower chamber. After 24 h, cell migration was examined. *n* = 3 per group. **d** CMT93 or LLC cells were seeded into the Matrigel-coated transwell upper chamber with WT or *Ndrg2−/−* TAMs, and TAMs pretreated with BAY-11-7082 were seeded into the lower chamber. After 24 h, cell invasion was examined. *n* = 3 per group. The results are presented as the mean ± SEM *p < 0.05, **p < 0.01, and ***p < 0.001

We used an in vitro co-culture system to investigate the effects of WT or *Ndrg2−/−* macrophages on cancer cell proliferation, migration, and invasion. In the wound-healing assay, WT or *Ndrg2−/−* macrophages were seeded in the upper chamber of the co-culture system, and CMT93 or LLC cells were seeded in the lower chamber. At 24 h after scratching, the wound had completely healed in the WT macrophage group compared with a 60% efficiency in the *Ndrg2−/−* macrophage group. WT macrophages promoted significant cancer cell migration. The use of BAY-11-7082-pretreated WT and *Ndrg2−/−* macrophages resulted in no differences between these two groups, suggesting that NF-κB pathway activation in *Ndrg2−/−* macrophages suppressed macrophage-mediated cancer cell migration (Fig. 7a). Consistently, in the colony-formation assay, conditioned medium from WT macrophages significantly promoted CMT93 cell colony formation compared with *Ndrg2−/−* macrophages (Fig. 7b).

Similar results were observed for migration and invasion in transwell assays. Macrophages have been shown to potentiate cancer cell migration and invasion. We evaluated the migration and invasion of CMT93 or LLC cells toward WT or *Ndrg2−/−* macrophages. *Ndrg2−/−* macrophages showed attenuated effects on the promotion of cancer cell migration and invasion. Additionally, the suppression effects of *Ndrg2−/−* macrophages were abolished by pretreating the macrophages with BAY-11-7082 (Fig. 7c, d), which suggested that the NF-κB pathway plays a pivotal role in inducing the *Ndrg2−/−* macrophage tumor-suppressor phenotype.

## Discussion

We initially identified NDRG2 as a new tumor suppressor^15^. After nearly 17 years of work, our laboratory and other groups have clearly shown that NDRG2 expression is decreased in multiple tumor tissues, including CRC, glioma, and hepatoma^15–21^. A large volume of data has shown that NDRG2 can inhibit cancer cell proliferation^15,20,22,23^. A recent study reported that NDRG2 is a PTEN-binding protein that recruits protein phosphatase 2A (PP2A) to PTEN^24^. Downregulation of NDRG2 in adult T-cell leukemia-lymphoma (ATLL) enhances PTEN phosphorylation and promotes the PI3K-AKT pathway. Researchers have further demonstrated that NDRG2 expression can downregulate the NF-κB pathway by inhibiting AKT in ATLL^25^. NDRG2 is also crucial in poorly differentiated CRCs. We recently demonstrated that NDRG2 can induce CRC cell differentiation by suppressing E3 ligase Skp2 activity. However, its role in tumor microenvironments, especially in TAMs, has not been illustrated.

In the present study, we aimed to gain a better understanding of the role of NDRG2 in the tumor microenvironment during the liver cancer metastasis process, hoping to find a new therapeutic target or prognostic biomarker for this life-threatening malignancy.

By establishing a liver metastasis model in WT and *Ndrg2−/−* mice, we found that loss of *Ndrg2* in the microenvironment significantly inhibited liver cancer metastasis. A similar phenotype has been observed in a subcutaneous tumor model in *Ndrg1−/−* mice. Kosuke et al. found that *Ndrg1−/−* mice have reduced tumor growth and cancer cell angiogenesis, accompanied by decreased infiltration and attenuated differentiation of TAMs^26^. In contrast, our flow cytometry analysis revealed that the *Ndrg2−/−* mice liver metastasis site accumulated a greater proportion of TAMs and a significantly greater proportion of M1-like TAMs.

Using BM transplantation together with macrophage and cancer cell mixed culture experiments, we confirmed that BMDMs were responsible for the suppression phenotype of the *Ndrg2−/−* liver microenvironment. Liver resident macrophages, KC, have also been reported to be pivotal immune cells that infiltrate the liver metastatic area^27^. Kupffer cells may directly kill cancer cells through secretion of TNF-α and ROS and phagocytose living cancer cells in a Dectin-2-dependent manner to prevent liver metastasis^27,28^. In contrast, KC have also been reported to have a protumorigenic effect by producing chemokines that contribute to extracellular matrix remodeling and angiogenesis^29–31^. We observed that the proportion of KC in *Ndrg2−/−* mice increased significantly in a liver cancer metastasis model compared with WT mice. However, as shown in Fig. 3c, in the metastasis model established in WT_BM_ → *Ndrg2−/−* and WT_BM_ → WT BM transplantation mice, we found that WT_BM_ → *Ndrg2−/−* mice showed significantly improved cancer metastasis, indicating that without the participation of BM-derived *Ndrg2−/−* cells, *Ndrg2−/−* KC together with other components of the *Ndrg2−/−* liver microenvironment did not exhibit a suppression phenotype but instead showed a significant tumor-promoting function. In addition, we observed tumor growth inhibition with *Ndrg2−/−* mice not only in the liver metastasis model but also in the subcutaneous inoculation model (Supplement Fig. 1b), suggesting that it was not the liver-specific components that predominantly contributed to the suppression phenotype. Collectively, *Ndrg2−/−* KC might not contribute to the liver tumor suppression microenvironment, and even if they participated partially, they were not as important as BMDMs. The mixed culture of macrophages and cancer cells demonstrated that the tumor-promoting function of *Ndrg2−/−* macrophages was diminished compared with WT macrophages. In addition, *Ndrg2−/−* macrophages re-educated recruited WT macrophages toward an M1-like phenotype.

A previous study reported that NDRG2 can inhibit IL-10 expression, which plays an important role in tumor-associated immune response by modulating SOCS3 and STAT3^32^. Consistently, we found that IL-10, an important M2 marker, was upregulated in *Ndrg2−/−* macrophages, conflicting with the M1-like phenotype of *Ndrg2−/−* macrophages. However, considering the other markers, such as IL-1β, IL-12, CD86 etc., tested using q-PCR, ELISA and flow cytometry, we confirmed that as a whole *Ndrg2−/−* macrophages tended to have an M1–like tumor-suppressor phenotype compared with WT macrophages.

Inhibition of *IKKβ* has been reported to promote the tumor-suppressing polarization of macrophages, whereas retention of *IKKβ* activation drives macrophages toward a tumor-promoting phenotype^33^. However, in established fibrosarcomas, inhibition of NF-κB pathway activation helps to drive the tumor-promoting phenotype of M2-like TAMs. Moreover, reactivation of the NF-κB pathway can shift M2 macrophages toward an M1-like phenotype^34,35^. All these findings suggest that NF-κB pathway activation in macrophages may play a distinct role in different tumor types. Our further experiments demonstrated that enhanced activation of the NF-κB pathway in *Ndrg2−/−* macrophages drives TAM polarization toward a tumor-suppressor phenotype. Inhibition of IκBα phosphorylation abolished the tumor-suppressor function of *Ndrg2−/−* macrophages in vitro.

Metastatic lesions in the liver frequently appear to undergo hypoperfusion with aberrant vascular formation. In light of the poor drug transport and uptake of tumor cells, macrophages are a superior therapeutic target considering their phagocytic features. Collectively, our study demonstrated that BMDMs play a pivotal role during liver cancer metastasis, and loss of *Ndrg2* inhibits this process by modulating macrophage polarization toward a tumor-suppressor phenotype by enhancing NF-κB pathway activation, indicating that macrophage NDRG2 may be a new therapeutic target for the treatment of liver metastasis.

## Materials and methods

### Cell culture and treatment

CMT93 murine colorectal carcinoma cells, murine LLC cells, and RAW 264.7 cells were purchased from the American Type Culture Collection (ATCC). Cells were cultured in DMEM with 10% FBS and 100 U per ml penicillin-streptomycin in a 5% CO_2_ humidified incubator at 37 °C.

Bone-marrow-derived macrophages were obtained as previously described. Briefly, 6-week-old mice were killed and soaked in 75% ethanol. Bone marrow cells were collected by flushing femurs and tibiae with PBS and cultured in DMEM with 10% FBS and 25 ng/ml GM-CSF for 6 days to obtain BMDMs.

Bone-marrow-derived macrophages and RAW 264.7 macrophages were stimulated with 100 ng/ml LPS and 20 ng/ml IFN-γ or 20 ng/ml IL-4 (PeproTech) separately for 24 h to obtain M1 or M2 macrophages. Conditioned medium from CMT93 cells was used to stimulate BMDMs and RAW 264.7 cells to obtain TAMs in vitro.

### Animal and liver cancer metastasis model

*Ndrg2* knockout (*Ndrg2*−/−) mice were generated by Shanghai Biomodel Organism Science & Technology Development Co., Ltd. and maintained on a C57BL/6J background. *Ndrg2* knock-in mice were generated by Beijing Biocytogen Co., Ltd. and maintained on a C57BL/6J background. All animals were raised under specific pathogen-free conditions. The mice were used in the experiments at 6−8 weeks of age. Wild-type and *Ndrg2*−/− mice were age-matched, and all mice were male. Animal experiments were approved by the Animal Experiment Administration Committee of the university.

For the liver cancer metastasis model, after the mice were anesthetized, a transverse incision in the left flank was made, and the spleen was exposed and separated into two parts. Next, 5×10^5^ CMT93-luciferase or LLC-luciferase cells in 100 µl of PBS were injected intrasplenically, after which this part of the spleen was removed to prevent splenic tumor formation; the other part was returned to the abdominal cavity to maintain its biological function^36^.

In the tumor cell and macrophage mixed culture model, 2×10^6^ CMT93 cells and 5×10^5^ macrophages were mixed in 100 μl of Matrigel and injected subcutaneously on the right side of the back of C57BL/6J mice. At the end of the experiments, the mice were killed, and tumors were weighed and analyzed using FACS or real-time PCR.

### In vivo bioluminescence imaging

The CMT93-luciferase and LLC-luciferase cells used for the liver metastasis model stably expressed firefly luciferase. On days 7 and 14 after model establishment, 200 µl of 15 mg/ml luciferin was intraperitoneally injected into anesthetized mice, and bioluminescence was examined 5 min after injection using an IVIS Lumina system. The bioluminescence was quantified based on the photon flux ratio^37^.

### Bone marrow transplantation

Donor BM was collected from 6- to 8-week-old WT or *Ndrg2*−/− mice by flushing femurs and tibiae with PBS. Eight-week-old recipients received a lethal 9 Gy irradiation dose. At 4 h after irradiation, 10^7^ donor BM cells were transplanted into recipients via tail vein injection. Six weeks after transplantation, recipients were used to establish the liver cancer metastasis model.

### Flow cytometry and cell sorting

Under deep ether anesthesia, the liver was perfused with Hanks Balanced Salt Solution (HBSS) via the portal vein. Immediately after perfusion, the liver was removed. Tumors were separated from the liver (with a margin of approximately 2 mm from the tumor edge) and minced with scissors. Liver specimens were incubated in 10 ml of HBSS containing 0.05% Type IV collagenase and 0.01 mg/ml DNase I (Sigma). The specimens were shaken for 40 min at 37 °C and then filtered through a stainless-steel mesh (70 µm), suspended in 33% Percoll solution and centrifuged for 15 min at 450 g at room temperature. After the red blood cells were lysed, the remaining cells were washed twice^38,39^.

For TAM magnetic sorting, single cells prepared as described above were incubated with Fc-blocker and then stained with biotin-conjugated anti-F4/80 Ab (ebioscience) and incubated with streptavidin magnetic beads (Biolegend). Positively labeled cells were collected using a magnet. For monocyte/macrophage magnetic sorting during the monocyte maturation process, biotin-conjugated anti-CD11b Ab (Biolegend) was used, followed by the above-described steps.

### ELISA and western blotting

For ELISA analysis, the indicated culture media were collected and tested using IL-10 and IL-12 ELISA kits.

For western blot analysis, after treatment, the cells were harvested and lysed with RIPA buffer (Boster). The protein concentration was determined using a BCA kit. Samples were separated on 12% SDS-PAGE gels and blotted onto PVDF membranes. Primary and secondary antibodies were incubated with the membranes at the indicated concentration.

### **Real-time PCR**

Total RNA was extracted from cultured or sorted macrophages using TRIzol reagent (Takara) according to the manufacturer’s instructions. cDNA was synthesized and analyzed via real-time quantitative PCR with SYBR Mix. The expression levels of the target genes were normalized to that of the control gene.

### **RT**^**2**^**Profiler PCR Array gene expression**

Total RNA was isolated from the CMT93 CM-induced WT and *Ndrg2−/−* TAMs using an RNeasy Mini Kit (catalog no. 74104; Qiagen). RNA quality was determined using a spectrophotometer and reverse-transcribed using an RT² First Strand Kit (catalog no. 330401; Qiagen). Complementary DNA was used for the real-time RT^2^ Profiler PCR Array Mouse Cancer Inflammation & Immunity Crosstalk (catalog no. PAMM-181Z; Qiagen) in combination with SYBR Green qPCR Mastermix (Roche)^40^.

### Immunohistochemistry

Mouse liver or tumors were fixed in formalin, embedded, sectioned, and stained with HE or the indicated antibody using standard immunohistochemistry methods.

### Wound-healing assay, clone-formation assay, and transwell assay

Co-culture systems were established using six-well culture plates and transwell inserts (0.4-mm pore, polycarbonate membrane; Costar). CMT93 cells (10^6^) were loaded into the lower chamber 12 h before co-culture, and BMDMs (10^6^) were seeded into the upper chamber of the culture system.

For the clone formation assay, 200 CMT93 or LLC cells were seeded in a six-well plate, followed by the addition of 3 ml of 5% FBS DMEM and 150 μl of TAM culture medium (concentrated from 1.5 ml) for 7 days.

For the transwell assays, inserts were coated with or without Matrigel for the invasion or migration assay, respectively. Next, 5×10^4^ CMT93 or LLC cells were seeded into the upper chamber and starved for 12 h before the experiments. TAMs were seeded into the lower chamber 12 h before the experiments.

For the NF-κB inhibition experiments, BMDMs were pretreated with 5 μm BAY-11-7082 (Sellck) for 4 h before co-culture, and the controls received an equivalent dilution with DMSO vehicle alone.

### Statistical analysis

The data are expressed as the mean ± SEM and were analyzed using an unpaired Student’s *t*test. *p* < 0.05 was considered statistically significant. All graphs and statistical calculations were performed using GraphPad Prism software.

## Electronic supplementary material

supplement figure legend

supplement figure 1

supplement figure 2

supplement figure 3

supplement table1
