# Supplementary material for: Loss of NDRG2 in liver microenvironment inhibits cancer liver metastasis by regulating tumor associate macrophages polarization
Source: Cell Death Dis. 2018 Feb 14;9(2):248. doi: 10.1038/s41419-018-0284-8 (PMC5833557; doi:10.1038/s41419-018-0284-8)
Supplement: Supplementary file 1 — supplement figure legend [file 41419_2018_284_MOESM1_ESM.docx]

***Table 1***. ***RT^2^ PCR array results***

Transcription factors related to 8 genes showing an upregulation of at least 2-fold in the PCR Array.

***Fig1.*** ***Loss of Ndrg2 inhibits tumor growth***

(a). Intrahepatic CMT93 cell injection model in WT and *Ndrg2-/-* mice. Bioluminescence was measured on days 7, 14, and 21 post-injection. Quantification of the photon flux ratio per mouse at each time point. (b). Subcutaneous injection of 2×10^^6^ CMT93 cells in WT and *Ndrg2-/-* mice. On day 21 post-injection, the mice were sacrificed, and the tumors were weighed. n=8 per group. (c). liver/body mass ratio of WT and *Ndrg2-/-* mice without tumor burden. n=6 per group. The results are presented as the mean±S.E.M. *p<0.05, **p<0.01, and ***p<0.001.

***Fig2.*** ***Proportion of infiltrated immune cells***

(a). immunostaining of infiltrated macrophages in WT and *Ndrg2-/-* mice liver metastasis area. (b).Proportion of T cells, B cells, NK cells, G-MDSCs, M-MDSCs and granulocytes infiltrated into the metastatic area. The results are presented as the mean±S.E.M. *p<0.05; ns indicates the difference was not significant.

***Fig3.*** ***RT^2^ PCR array***

(a). After treatment of WT and *Ndrg2-/-* BMDMs with CMT93 cell-conditioned medium, twenty-two genes showed upregulated expression greater than 1.5-fold in a Mouse Cancer Inflammation & Immunity Crosstalk real-time RT^2^ Profiler PCR Array. Genes that were upregulated more than 2-fold were considered significantly upregulated.
