# Supplementary figures and images for: Loss of NDRG2 in liver microenvironment inhibits cancer liver metastasis by regulating tumor associate macrophages polarization
Source: Cell Death Dis. 2018 Feb 14;9(2):248. doi: 10.1038/s41419-018-0284-8 (PMC5833557; doi:10.1038/s41419-018-0284-8)

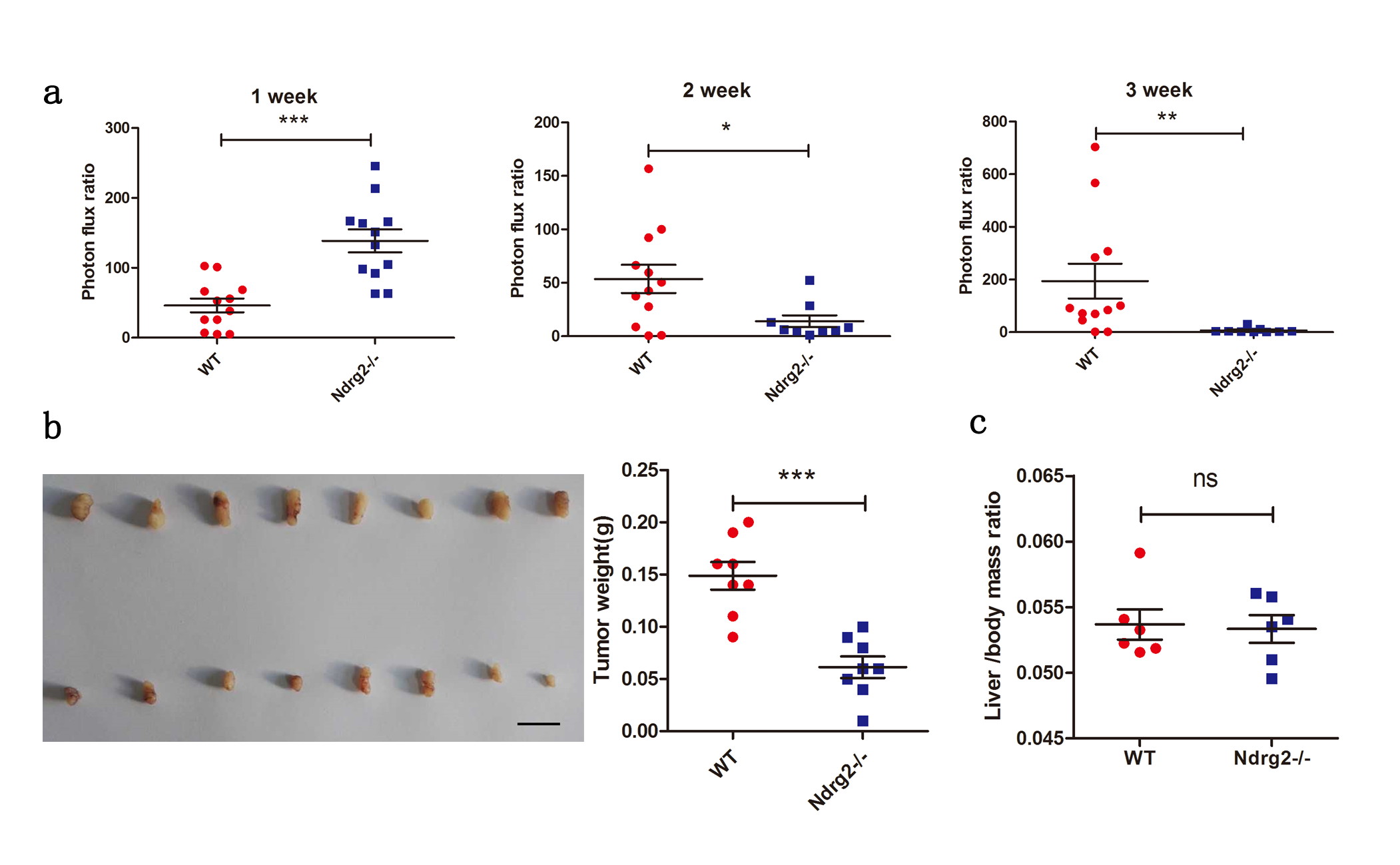

Supplement: Supplementary file 2 — supplement figure 1 [file 41419_2018_284_MOESM2_ESM.tif]

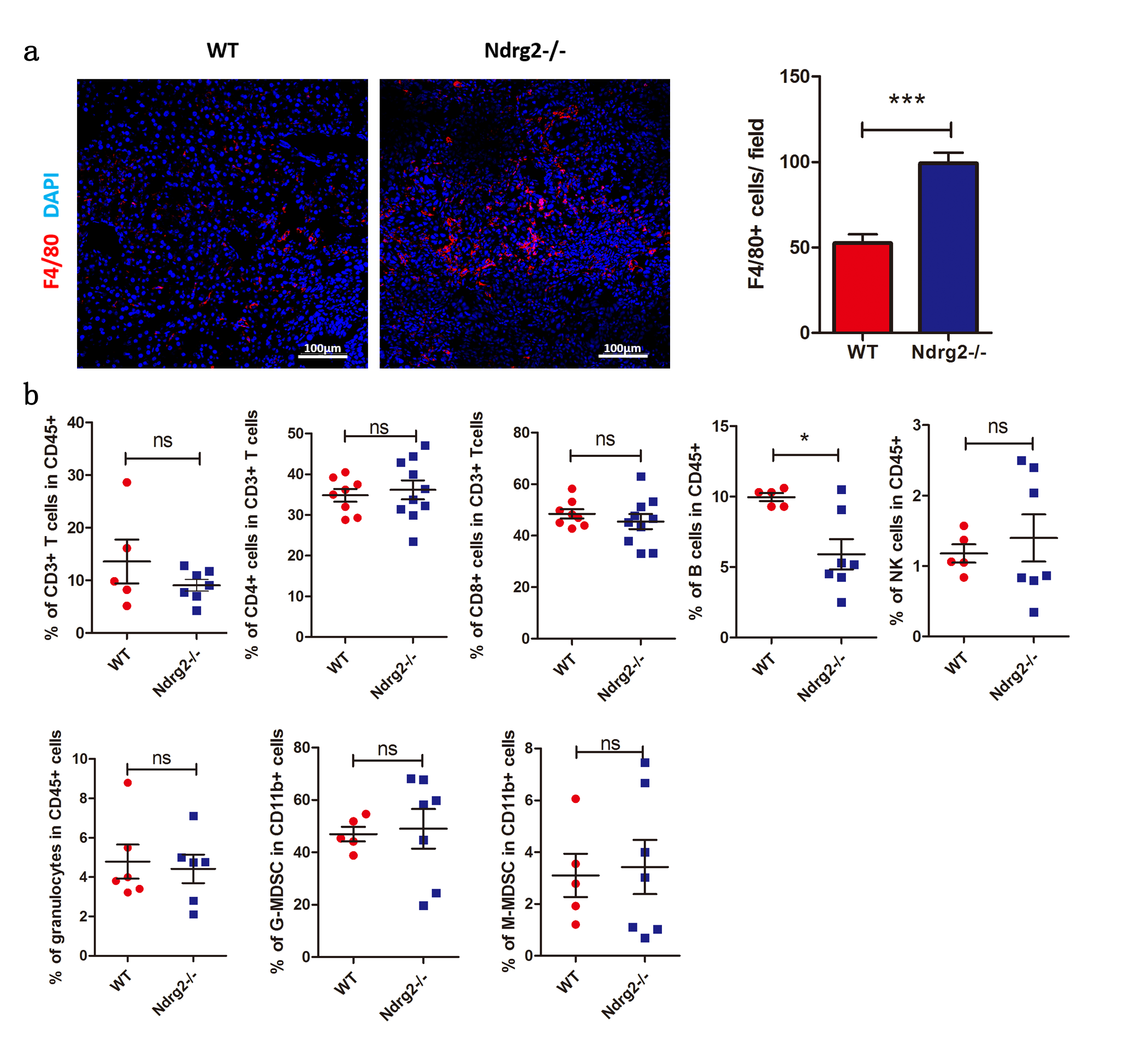

Supplement: Supplementary file 3 — supplement figure 2 [file 41419_2018_284_MOESM3_ESM.tif]

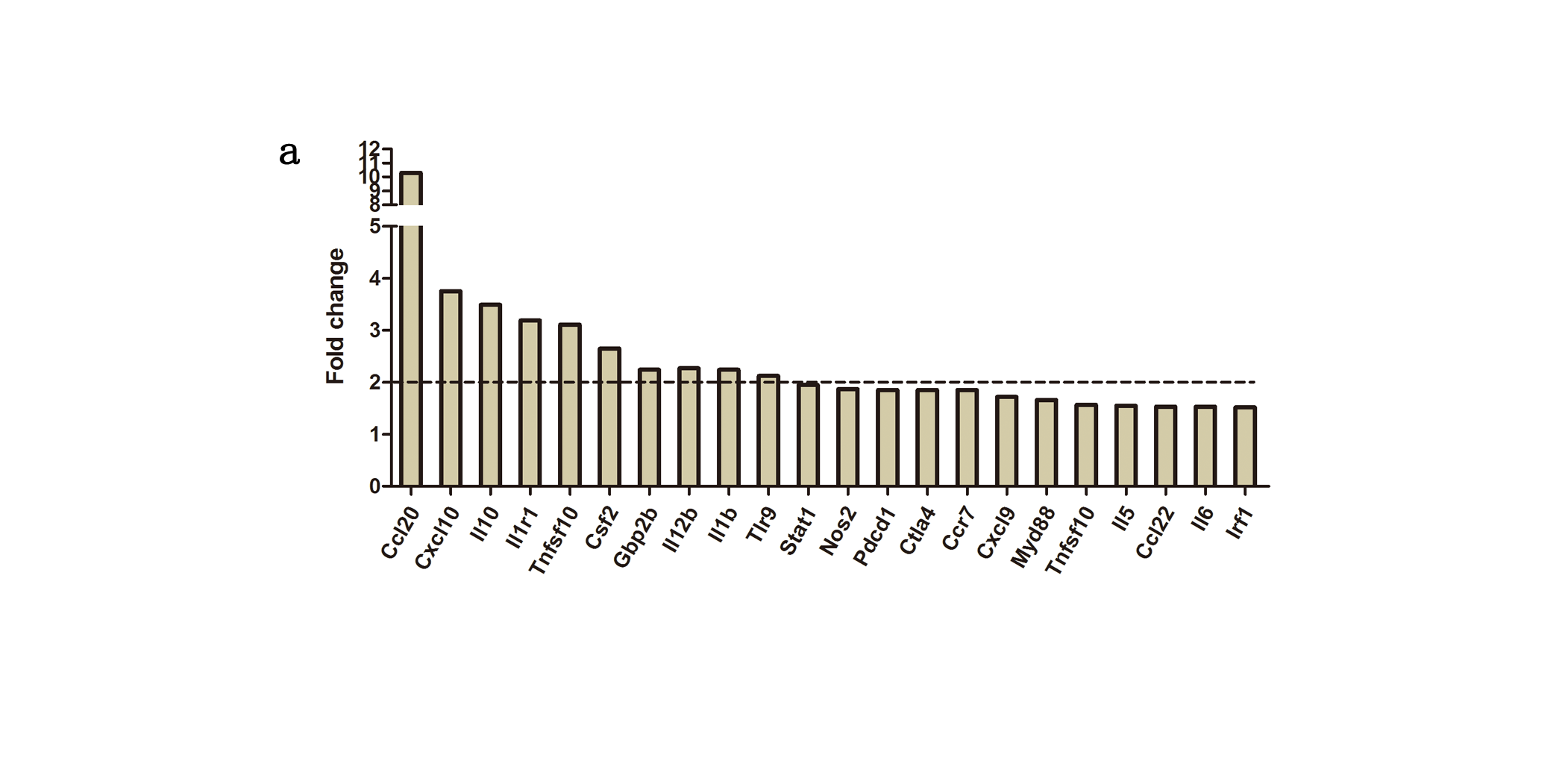

Supplement: Supplementary file 4 — supplement figure 3 [file 41419_2018_284_MOESM4_ESM.tif]
