# Supplementary material for: Loss of NDRG2 in liver microenvironment inhibits cancer liver metastasis by regulating tumor associate macrophages polarization
Source: Cell Death Dis. 2018 Feb 14;9(2):248. doi: 10.1038/s41419-018-0284-8 (PMC5833557; doi:10.1038/s41419-018-0284-8)
Supplement: Supplementary file 5 — supplement table1 [file 41419_2018_284_MOESM5_ESM.docx]

Supplement Table1.

***Transcription factors related to 2-fold-change genes***

| Gene | Fold Regulation | Transcription Factors |
| --- | --- | --- |
| Ccl20 | 10.30 | Cdc5, NF-kappaB1, NF-kappaB, NF-kappaB2, c-Rel,NF-kappaB1, RelA, NF-kappaB, Pax-5 |
| Cxcl10 | 3.75 | LCR-F1, NF-E2, NF-E2 p45, NF-kappaB1,NF-kappaB, NF-kappaB2, NF-kappaB1, RelA,NF-kappaB, c-Rel, NF-kappaB1, NF-kappaB,NF-kappaB2, c-Rel, NF-kappaB1, RelA, NF-kappaB,ISGF-3, IRF-1, IRF-2 |
| Il10 | 3.49 | TBP, aMEF-2, MEF-2, MEF-2A, RSRFC4, E47, Tal-1,Tal-1beta, ITF-2, PPAR-gamma1, PPAR-gamma2,ER-alpha, HTF, XBP-1, ATF6, C/EBPbeta |
| Il1r1 | 3.19 | Cdc5, Meis-1, Meis-1b, HOXA9, HOXA9B, RP58 |
| Csf2 | 2.65 | FOXD1, Pax-2, Pax-2a, Pax-2b, Nkx2-2, RREB-1,CP2, LUN-1 |
| Il12b | 2.27 | aMEF-2, MEF-2, MEF-2A, Nkx2-2, ATF-2, NRSF form1, NRSF form 2, NF-kappaB1, NF-kappaB,NF-kappaB2, NF-kappaB1, RelA, NF-kappaB |
| Il1b | 2.24 | TBP, TBP, TFIID, C/EBPbeta, c-Rel, RelA, NF-AT,NF-AT1, NF-AT2, NF-AT3, NF-AT4 |
| Stat1 | 1.95 | NF-YA, NF-YB, NF-YC, FOXO1, FOXO1a |
| Gzma | -7.65 | AML1a |
| Kitl | -5.01 | Pax-5, AhR, Arnt, IRF-7A |
| Csf3 | -2.59 | Arnt, USF-1, USF1, AREB6, SREBP-1a, SREBP-1b,SREBP-1c, SEF-1 (1), Elk-1, IRF-7A, c-Ets-1, AREB6,NF-AT, NF-AT1, NF-AT2, NF-AT3, NF-AT4, Bach1,c-Jun, AP-1, c-Fos, FosB, Fra-1, JunB, JunD, Bach2,c-Rel, NF-kappaB1, RelA, NF-kappaB, p53, Pax-5,CP2, AREB6, POU2F1, POU2F1a, Oct-B1, oct-B2,oct-B3, POU2F2, POU2F2 (Oct-2.1), POU2F2B,POU2F2C, POU2F1, POU2F1a, POU2F1b,POU2F1c, C/EBPalpha, POU3F2, POU3F2(N-Oct-5a), POU3F2 (N-Oct-5b), STAT5A |
| Cxcr1 | -2.47 | CUTL1, Nkx3-1, Nkx3-1 v1, Nkx3-1 v2, Nkx3-1 v3,Nkx3-1 v4, Nkx2-5, CUTL1 |
| Foxp3 | -2.18 | FOXO1, FOXO1a, TBP, TBP, TFIID, SEF-1 (1),CUTL1, Meis-1, Meis-1b, HOXA9, HOXA9B, MZF-1,C/EBPalpha, AML1a, PPAR-gamma1, PPAR-gamma2,GATA-1, AML1a, SRY, Ik-1 |
| Il13 | -2.01 | HSF1 (long), HSF1short, RelA, AML1a, GATA-1,Lmo2, Bach2, CREB, deltaCREB, TBP, TFIID |
